# Estimating the impact of label design on reducing the risk of medication errors by applying HEART in drug administration

**DOI:** 10.1007/s00228-024-03619-3

**Published:** 2024-01-29

**Authors:** Carlos Aceves-Gonzalez, Angela Caro-Rojas, John A. Rey-Galindo, Luz Aristizabal-Ruiz, Karen Hernández-Cruz

**Affiliations:** 1 Universidad de Guadalajara, Guadalajara, Mexico; 2 International Society of Pharmacovigilance, London, UK; 3 Vitalis Lab, Bogota, Colombia

**Keywords:** Label design, Medication errors, HEART

## Abstract

**Abstract:**

Medication errors are one of the biggest problems in healthcare. The medicines’ poor labelling design (i.e. look-alike labels) is a well-recognised risk for potential confusion, wrong administration, and patient damage. Human factors and ergonomics (HFE) encourages the human-centred design of system elements, which might reduce medication errors and improve people’s well-being and system performance.

**Objective:**

The aim of the present study is twofold: (i) to use a human reliability analysis technique to evaluate a medication administration task within a simulated scenario of a neonatal intensive care unit (NICU) and (ii) to estimate the impact of a human-centred design (HCD) label in medication administration compared to a look-alike (LA) label.

**Method:**

This paper used a modified version of the human error assessment and reduction technique (HEART) to analyse a medication administration task in a simulated NICU scenario. The modified technique involved expert nurses quantifying the likelihood of unreliability of a task and rating the conditions, including medicine labels, which most affect the successful completion of the task.

**Results:**

Findings suggest that error producing conditions (EPCs), such as a shortage of time available for error detection and correction, no independent checking of output, and distractions, might increase human error probability (HEP) in administering medications. Results also showed that the assessed HEP and the relative percentage of contribution to unreliability reduced by more than 40% when the HCD label was evaluated compared to the LA label.

**Conclusion:**

Including labelling design based on HFE might help increase human reliability when administering medications under critical conditions.

## Introduction

Unsafe medication practices and medication errors are known to be leading causes of avoidable harm in healthcare systems worldwide. Medication errors can cause mild or severe damage, disability, and even death and are estimated to cost $42 billion globally annually. The scale and nature of this harm differ between countries, with a higher impact on those patients living in low-income countries [1]. These circumstances have led to the World Health Organization’s (WHO) Third Global Patient Safety Challenge: Medication Without Harm, which aims to reduce the global level of severe, avoidable harm related to medications by 50% between 2017 and 2022.

Although medication errors could arise in all drug management processes, including prescription, preparation, dispensation, administration, and monitoring, current research suggests that most medication errors occur at the administration stage [2]. Intravenously administered drugs have been associated with the highest frequencies of medication administration errors [3] and the most severe consequences for patients. Errors may arise due to the complexity of administering intravenous medication as a multi-step process involving specific administration devices, information systems, and several healthcare professionals with different tasks and competencies [4]. Likewise, given their complexity and patient characteristics, current research suggests medication safety may be critical in intensive care units (ICUs) [5] and, in particular, in paediatric and neonatal ICUs (PICUs and NICUs) [6, 7]. For instance, in a UK study examining 441 medication errors in hospitalised children over 2 years, Wilson et al. [8] found that medication errors in PICUs occurred seven times more frequently than in other paediatric inpatient units.

Several studies and systematic reviews have explored the factors contributing to medication errors. For instance, in an early and seminal study, Chapanis and Safren [9] found that the causes of medication safety incidents fell into five categories: (i) non-compliance with required checking procedures, (ii) misreading or misinterpretation of written communications, (iii) transcription errors, (iv) misplaced medicine tickets at the ticket box, and (v) miscalculation errors. In a recent systematic review of the systemic causes of in-hospital intravenous medication errors, Kuitunen et al. [4] identified that insufficient actions to secure the safe use of high-alert medications, lack of knowledge of the drug, calculation tasks, failure in double-checking procedures, and confusion between look-alike medications are leading systemic causes for medication errors affecting more than one phase of the medication process.

Medication label design is often a contributing factor to medication errors. Research has shown that one-third of reported medication incidents may be due to confusion over packaging and labelling [10]. Over the last two decades, different international reports such as the Institute of Medicine (IOM) report, To Err is Human: Building a Safer Health System [11], have suggested that drugs may be prone to error in use due to sound-alike or look-alike names, unclear labelling, or poorly designed packaging. Poor labelling design can contribute to medication errors by making it difficult for end users to identify and understand critical safety information [12]. In an integrative review, Borradale et al. [13] found that packaging and labelling design is the most commonly identified factor contributing to misreading injectable medications; the specific features named as problematic were as follows: look-alike injectable medications, similar colours, small text, lack of colour contrast on ampules, embossed information on plastic ampules, trade name prominence, and the design of ampule/vial labels. Literature suggests that pharmaceutical companies do not consider design for safety their responsibility [14] and assume that healthcare staff are those who should prevent medication errors [15]. As a result, several international organisations have published recommendations on the optimal design of medicines labels [12, 16, 17]. However, although those recommendations are based on human-centred design principles, minimal evidence supports their adoption [18].

Human factors and ergonomics (HFE) encourages the human-centred design of system elements to improve people’s well-being and system performance. It has been applied for patient safety improvement in different healthcare domains [19, 20]. Indeed, the WHO’s Global Patient Safety Action Plan 2021–2030 identified HFE as a critical strategy for building highly reliable health systems and organisations that protect patients from daily harm [21]. Recently, there has been an increasing interest in applying HFE in pharmacy to promote the human-centred design of systems to support individuals and teams performing medication-related work [22, 23]. Some scholars suggest the need to invest in healthcare HFE skills and professionals for medication safety improvement [24], integrate HFE specialists into multidisciplinary teams to improve intravenous medication safety [25], and embed HFE and patient safety education in pharmacy curricula [26]. Also, efforts have been made to integrate HFE in researching the design of medication labels [27].

Human error identification (HEI) methods fall within the field of HFE [28]. They could provide a valuable framework for analysing and reducing risks in healthcare [29], including medication safety. These prospective human error methods are used to identify all possible types of errors that may occur during specific tasks and suggest design solutions that can be applied in advance [30] to improve reliability and safety [31]. These methods can be used to identify or quantify errors. An example of a human error identification method is the Systematic Human Error Reduction and Prediction (SHERPA) [32], which has been used for the prospective analysis of errors in dispensing [33] and administering medicines [34].

Error quantification methods are used to determine the numerical probability of error occurrence [28]. The human error assessment and reduction technique (HEART) [35] is an HRA method that attempts to predict and quantify the likelihood of human error or failure within complex systems. Although HEART was developed within the nuclear power and chemical process industries [28], it is intended to be applicable to different sectors [36]. Literature on patient safety highlights the advantages of transferring and applying HRA methods to healthcare services [37]. There is evidence of theoretical contributions to the use of the HEART method in healthcare (Lyons et al. [31]; Lyons [45]), as well as its application in areas such as surgery [38, 39], radiotherapy [36, 40], and blood transfusion [41].

This study builds on assessing a design intervention of injectable medicine labels resulting from a programme for patient safety developed by a pharmaceutical company in Colombia [42]. After a risk analysis of medicine labels, Garnica and Aristizabal [42] identified some company products as high risk due to label design and similarity (look-alike medications, similar colours, small text, and lack of colour contrast on ampules, among others). A new labelling design was developed under the human-centred design (HCD) guidelines for safer medication provided by the Institute for Safe Medication Practices [16] and the National Patient Safety Agency [17]. Our study used a simulated scenario of a NICU to estimate the impact of the previous label design (*look-alike (LA)* label) versus the new label design (HCD label) on human reliability using HEART.

To the best of the authors´ knowledge, the current study is the first proactive analysis of HRA in the drug administration process and the influence of label design using HEART. Therefore, the aim of the present study is twofold: (i) to use a human reliability analysis technique to evaluate the task of medication administration within a simulated scenario of a NICU and (ii) to estimate the impact on the human reliability of a human-centred design label in medication administration compared to a label without those design characteristics. To this end, the following Research Questions are posed:

- rq1: What is the likelihood of human unreliability in a medication administration task within the simulated scenario of a NICU?
- rq2: What is the estimated impact on the human reliability of a human-centred design label in medication administration compared to a label without those design characteristics?

## Methodology

### Participants

A convenience sample of eight nurses with experience in critical hospital services (e.g. emergency, surgery, or intensive care unit) was recruited to participate in expert groups. Nurses were invited to act as substantive experts [43] since they must sufficiently know the process under analysis. Invitations to participate in the study were sent to groups of nurses working in public and private hospitals in Bogota, Colombia. Participants were required to have experience in medication administration either in public or private hospitals and to be working or to have worked in one of the critical services mentioned above. Of the nurses who showed interest in participating, the researchers selected those with more years of experience in medication administration and working in critical services. All the participants signed an informed consent before their participation.

### Simulated scenario

Simulated scenarios have been used previously for understanding the way to minimise risk in the use of medicines [18, 44]; it helps in the identification of cognitive processes, especially when it is not possible to measure them in real-world situations or for characterising fatal events with low incidence and report (such as adverse events by medication errors).

For this study, a simulated scenario was developed under a realistic approach in one of the critical services previously identified by the research team. The simulated scenario was designed to provide enough elements and details for participants to mentally engage in the case as they would in real-life care. This scenario was presented to the participants as described below:

‘You work in a public hospital’s Neonatal Intensive Care area, which has ten beds, of which eight are occupied; you are assigned to the afternoon shift (1 to 7 pm). It is now 3:50 pm. Your responsibilities include administering medication to the eight patients. You also must feed them and do other routine tasks. Two nursing assistants with five and three years of experience work the same shift with you. As a nurse, you must administer two prescribed medications for the patient identified as H/Maria Perez at 4:00 pm. Your task includes picking up the medications from the NICU stock box, taking them to the medication preparation area, reconstituting them, diluting them in a large volume liquid bag and proceeding with the infusion pump administration.’

### Medication labels assessed in this study

**Fig. 1:**
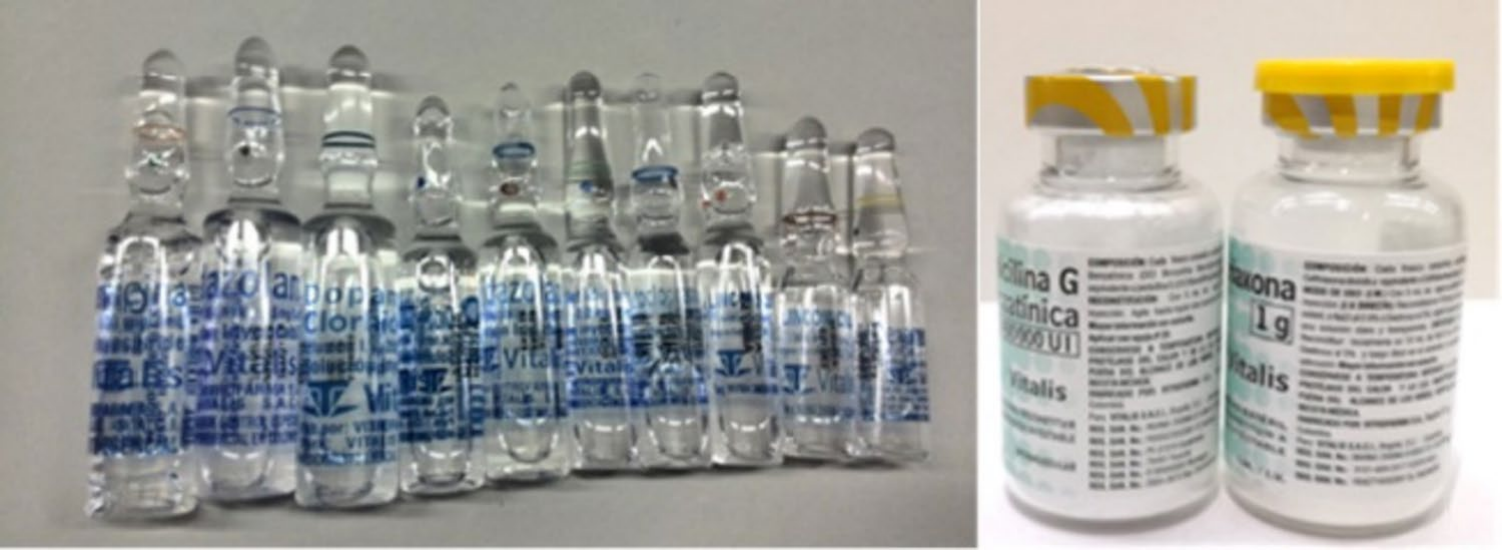
Medicines with look-alike labels (ampoules and vials)

**Fig. 2:**
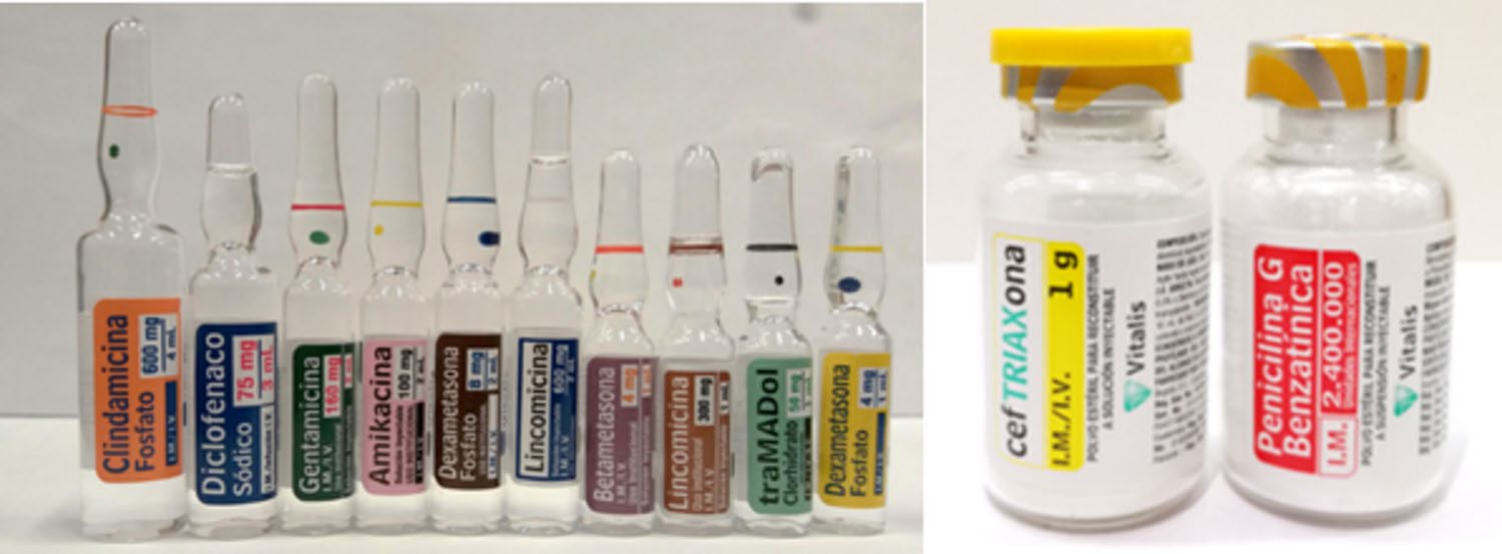
Medicines with human-centred design labels (ampoules and vials)

Label designs included in this study are shown in Fig. 1 (*look-alike labels*) and Fig. 2 (human-centred design labels). The HCD label design included colour differentiation between products (inter-class), using a white background, avoiding dangerous abbreviations, tall-man lettering, and vertical text to allow better readability [42].

### Generating the hierarchical task analysis (HTA)

Hierarchical task analysis (HTA) is the most popular task analysis method and perhaps the most widely used of all available HFE methods [28]. Although HTA is not required for HEART application [40, 45], it is suggested that a specific type of task description may support the understanding of a critical task [28, 45]. Therefore, in this study, we used an HTA to give participants a visual overview of the task under investigation. The lead author developed the HTA for the medication administration process based on the technique described by Lane et al. [34] and the description made by a pharmacist with 15 years of experience on medication safety (Second author).

This HTA was then shown to the participants of the expert group, who were then asked to discuss and validate the tasks included in the HTA. Participants identified some tasks to be added to the final HTA of medication administration. The HTA was also used by the experienced nurses and the research team to identify the specific tasks where the label design is critical for the administration process.

**Fig. 3:**
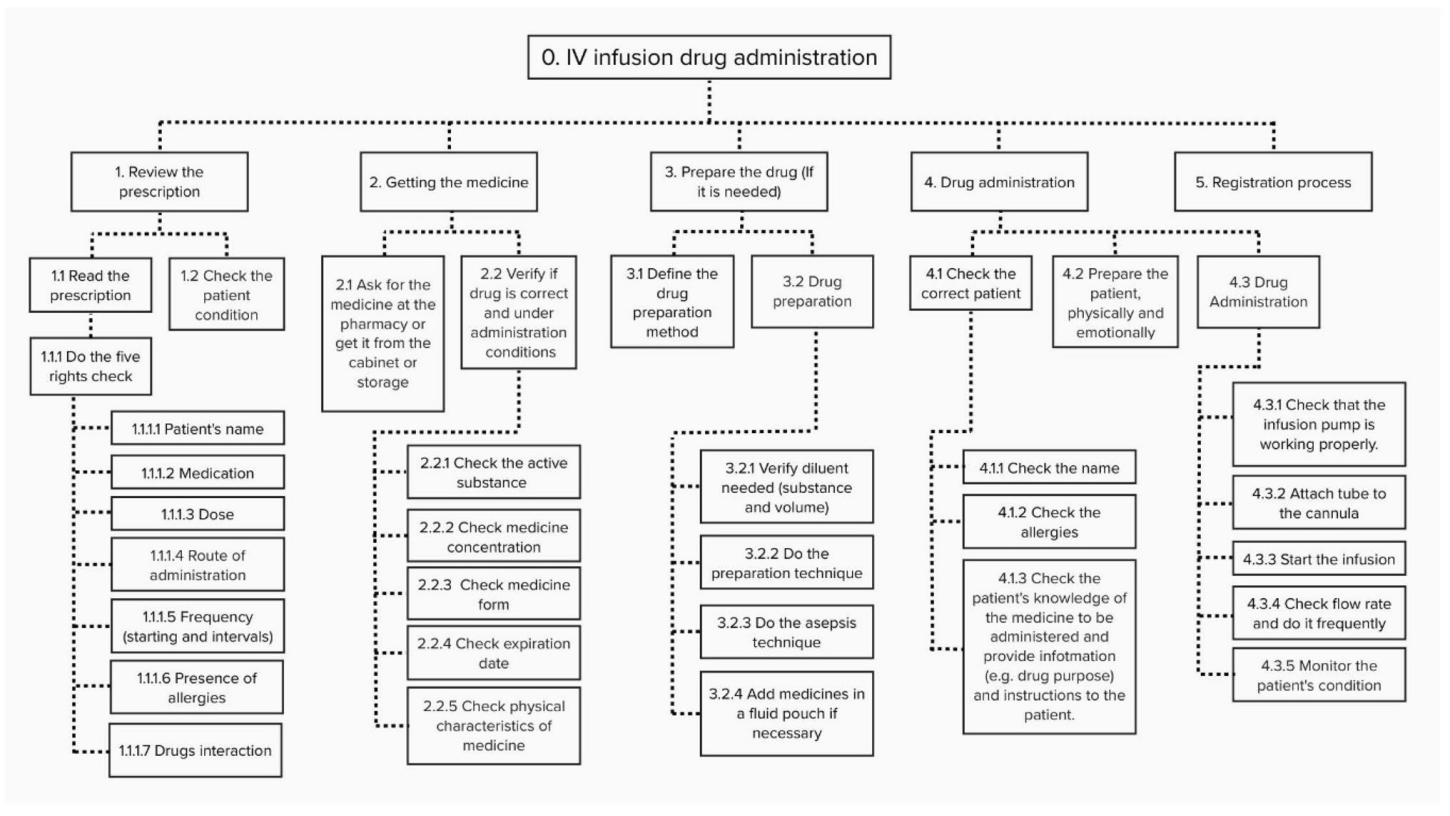
HTA for IV infusion drug administration task

The hierarchical task analysis (HTA) for the IV infusion medication administration task is shown in Fig. 3. The top-level goal of the system is to administer IV drugs to the patient. The tasks necessary are listed as tasks 1 to 5 on the following hierarchy level. These activities are further broken down into operations at the lower levels.

### Human error assessment and reduction technique (HEART)

The human error assessment and reduction technique (HEART) was developed by Williams [35] to assist engineers in evaluating human reliability on system performance. It was designed to be a simple, easily understood, systematic, and repeatable tool to identify the significant influences on human performance. The method is based on the general idea that each task in life poses a probability of failure, and for each task, there are varying levels of error producing conditions (EPCs) that may influence human reliability [46].

Regarding the validation of HEART, Kirwan et al. [47] describe the validation of three techniques: HEART, THERP, and JHEDI. The results showed a significant overall correlation of all estimates with the known valid values, 23 significant individual correlations, and a general precision range of 60–87%, with an average of 72%. The highest precision rating associated with the HEART technique was 76.67%. According to Kirwan et al. [47], the results demonstrate the empirical validity of the three methods.

**Fig. 4:**
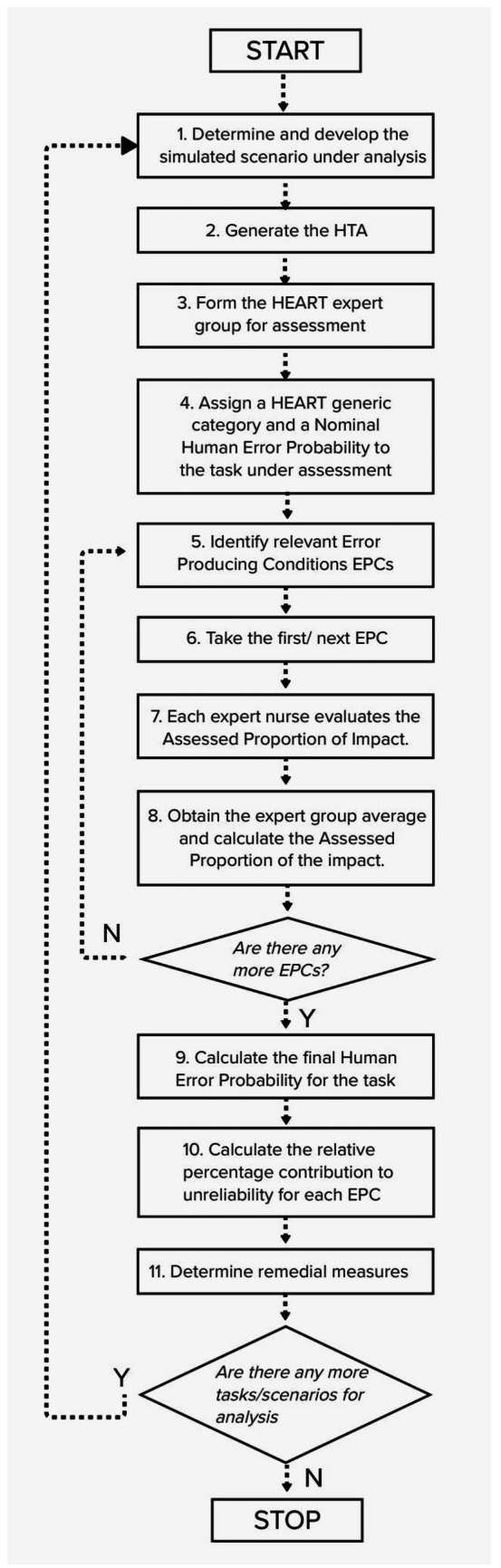
Modified HEART method

The steps for the modified HEART method are delineated in Fig. 4 (based on Chadwick and Fallon [40]; Stanton et al. [28]; Williams [35]) and described in the ‘Assessment procedure’ section.

### Assessment procedure

The procedure to undertake the human reliability assessment of an IV medication administration task and to estimate the impact on patient safety of a human-centred design label compared to a look-alike label was as follows:

- *Step 1. Determine* *and develop the simulated scenario under analysis*. The scenario was developed as described previously in the ‘Simulated scenario’ section.
- *Step 2. Generate* *the hierarchical task analysis (HTA) for IV drug administration*. The HTA was generated as described in the ‘Medication labels assessed in this study’ section.
- *Step 3. Form the HEART expert groups for assessment.* Eight experienced nurses formed two expert groups, four in each group. They helped to revise and validate the HTA as described in the ‘Generating the hierarchical task analysis (HTA)’ section. In addition, they were asked to actively select the HEART generic category (step 4), identify relevant EPCs (step 5), and assess the proportion of the impact of each EPC (steps 6 and 7).
  The first and second authors led the group sessions, acting as facilitators providing support and clarification to the participants when required, but without participation in selecting or assessing the tasks or EPCs.
- *Step 4. Assign a HEART generic category and a nominal human error probability to the task under assessment.* In the original HEART method, this step is performed by a single expert evaluator. However, this has been criticised as being too highly dependent on the expert evaluator [40, 47]. Chadwick and Fallon [40] point out that this is particularly relevant in healthcare, where HFE specialists or risk assessors might not have significant first-hand ‘sharp end’ experience in highly specialised healthcare domains. In contrast, healthcare staff are typically expert ‘operators’ in treatment processes. Therefore, our study involved experienced nurses assigning a generic probability to the task using the consolidated version of generic HEART categories [48] (Appendix 1). A similar HEART method modification was done in a previous study in the healthcare sector [40].
- *Step 5. Identify relevant error producing conditions (EPCs).* These EPCs (Appendix 2) are the factors that influence human reliability when performing a given task. In our study, the experienced nurses were asked to identify the EPCs that might significantly influence nurses’ performance when administering the medication in the simulated scenario. In addition, participants were invited to identify any EPCs that might help assess the influence of label design. This study used the consolidated version of HEART EPCs (Williams and Bell [46]).
- *Step 6. Select the EPC and Step 7. Evaluate the assessed proportion of impact.* The participants select EPCs potentially involved in the scenario (e.g. high workload when administrating the IV medicine) and calculate the assessed proportion of impact. According to the method, experts must assess the proportion of each EPC’s impact on the task being evaluated. This involves providing a rating between 0.1 and 1.0 (where 0.1 represents lower risk, and 1.0 represents higher risk) for each EPC. Participants were asked to evaluate this according to their perception of the impact of the specific EPC on medication safety in the analysed scenario. After each score, nurses could discuss and sustain the reasons behind the value assigned to each EPC.
  Furthermore, in this study, participants evaluated the assessed proportion of impact of the selected EPCs related to medication labels, both LA and HCD labels. The nurses had time to interact with ampoules and vials before providing their response for the proportion of the impact of each type of label. LA and HCD labels were presented alternately in each group; thus, group one rated the LA label first and then the HCD label, and the nurses in group two did it inversely.
- *Step 8. Obtain the expert group average and calculate the assessed proportion of effect*. The results from each EPC are averaged by the research team, considering participants of both groups. The next step includes calculating the assessed proportion of the impact of each selected EPC.
- *Step 9. Calculate the human error probability for the task and Step 10. Calculate the percentage contribution to unreliability for each EPC*. The research team calculates the overall human error probability and the relative percentage contribution to unreliability based on the equation provided by the method. The percentage contribution to unreliability allows rating the EPCs based on their gross effect on the HEP.

## Results

Eight female nurses participated in the expert groups. Table 1 summarises participants’ characteristics regarding their roles and experience. Participants were organised into two sessions. Each session was attended by four nurses and lasted approximately 2 h.

**Table 1:** Characteristics of experienced nurses who participated in the study

Nurses’ roles	Experience in medication administration	Work in a critical service	Experience in critical services
Chief of Nursing	More than ten years	Intensive care unit	Six years
Chief of Nursing Department	More than ten years	Intensive care unit	Ten years
Chief of Nursing	More than ten years	Emergency room	15 years
Leader nurse of safety programs	From 7 to 10 years	Emergency room	Four years
Independent consultant in patient safety	From 7 to 10 years	Emergency room	Four years
Nurse	From 7 to 10 years	Intensive care unit	Seven years
Nurse	More than ten years	Emergency room	Three years
Chief of nursing surgery services	From 7 to 10 years	Surgery room	Two years

### Selection of the HEART generic category

The HEART generic category selected by the nurses was Category Task G, described as: ‘Completely familiar, well-designed, highly practised, routine task occurring several times per hour, performed to highest possible standards by highly motivated, highly trained and experienced person, totally aware of implications of failure, with time to correct potential error, but without the benefit of significant job aids.’ (See Appendix 1).

However, the participants raised some observations about this task description, so some suggested generic task C, described as: ‘Complex task requiring a high level of understanding and skill’. They noted that administering medication in the scenario studied did not allow sufficient time to correct a potential error, and the staff might not always be highly motivated. Finally, participants agreed to select task G and suggested that those elements of the generic task description should be considered when estimating the potential risk of this task.

This Category has a nominal human error probability of NHEP = 0.002 [49].

### Selection of EPCs and determining the assessed proportion of the impact

The EPCs that the nurses selected as having the most significant influence on task performance in the simulated scenario and a rationale for their selection are provided in Table 2. It is essential to mention that the nurses also recognised additional EPCs that they considered to affect the completion of the administration task. It must be noted that this part of the process needed more attention and time to make the appropriate choice of the EPCs involved in the task under analysis. In addition, it was occasionally necessary to provide examples to fully understand each EPC definition and how it may potentially influence human reliability.

**Table 2:** Relevant EPCs chosen by participants

EPC	EPC description	Rationale for use
2	A shortage of time available for error detection and correction	Given the workload of the nursing staff due to the number of patients to be seen and the additional administrative tasks to be performed, there is little time to check for errors
6	A mismatch between an operator’s model of the world and that of a designer – also known as Model Mismatch	There is often poor interface design, particularly in the labelling of medicines. Designers do not consider the needs of nurses and pharmacists
17	Little or no independent checking or testing of output	Due to staff shortages and workload, it is often not possible to carry out any independent verification of results
32	Inconsistency of meaning of displays and procedures	The design of displays (labelling) often is inconsistent with the type of medicine, level of risk, or the needs of those administering the medicine. Poorly designed displays
39	Distraction/Task Interruption	Due to the presence of family members and other professionals in the area, distractions/interruptions might occur frequently

Participants of the expert groups chose EPCs 6 and 32 as adequate to analyse the label design. We decided to run the analyses with both EPCs for the label design to explore different scenarios. HEART calculations were estimated in this study using label design with both EPCs. The average assessed proportion of affect result for each identified EPC is presented in Table 3. EPCs 6 and 32 values are shown for the LA and HCD labels, respectively. The HEART technique suggests a rating between 0.1 and 1.0 for each EPC (where 0.1 represents lower risk, and 1.0 represents higher risk). It can be noted that a reduction of up to 0.6 on the average assessed proportion of impact with both EPCs when the HCD label was evaluated compared to the LA label.

**Table 3:** Average assessed proportion of impact results of each EPC

Participant code	EPC:2	EPC:6^a^		EPC:17	EPC:32^a^		EPC:39
		LA label	HCD label		LA label	HCD label	
EG1_1	1.0	0.9|	0.3	0.9	1.0	0.1	0.9
EG1_2	0.9	1.0	0.3	0.7	1.0	0.5	0.8
EG1_3	0.9	0.8	0.1	0.8	1.0	0.3	1.0
EG1_4	0.8	0.8	0.2	0.9	0.9	0.1	0.9
EG2_1	0.9	0.7	0.3	0.9	0.7	0.2	0.9
EG2_2	0.8	0.8	0.2	1.0	1.0	0.4	0.8
EG2_3	1.0	0.8	0.2	1.0	1.0	0.3	1.0
EG2_4	0.9	0.8	0.2	0.7	0.7	0.5	0.9
The average assessed proportion of the impact	0.9	0.82	0.22	0.92	0.91	0.3	0.9

^a^These EPCs show the difference between the values assigned by the participants for the LA and HCD labels

### HEART calculations of assessed human error probability (AHEP)

After calculating the assessed EPC impact for each of the chosen EPCs, the assessed human error probability for the IV medication administration task was calculated using the equation provided by Heart [35]. Table 4 shows a reduction in the AHEP when using the EPC 6 for label design, from 1.33 to 0.51 when was evaluated the LA and HCD labels, respectively. If it is considering that a total probability of failure can never exceed 1.00, and the probability of failure has to be assumed to be 1.00 when the multiplication of factors takes the value above 1.00 [35]; therefore, there was a reduction from 1.00 to 0.51 in the AHEP when the HCD label was assessed.

**Table 4:** HEART calculations of AHEP—label design assessed as the EPC 6

Generic task type = G Nominal human error probability = 0.002			
EPC	HEART effect multiplier	The assessed proportion of impact	Assessed EPC effect
2. A shortage of time available for error detection & correction	11	0.9	((11–1) × 0.9) + 1 = 10
17. Little or no independent checking or testing of output	3	0.86	((3–1) × 0.86) + 1 = 2.7
39. Distraction/Task Interruption	4	0.9	((4–1) × 0.9) + 1 = 3.7
6. A mismatch between an operator’s model of the world and that of a designer	8	0.8 (LA label) 0.22 (HCD label)	((8–1) × 0.8) + 1 = 6.6 ((8–1) × 0.23) + 1 = 2.5
Total assessed EPC effect	LA label	= (10 × 2.7 x × 3.7 × 6.6) = 664.22	
	HCD label	= (10 × 2.7 × 3.7 × 2.5) = 255.63	
**Assessed human error probability**	**LA label = (0.002)** × 664.22 = 1.33* **HCD label = (0.002)** × **255.63 = 0.51**		

Table 5 shows the AHEP results when using the EPC 32 for assessing label design. A reduction in this value from 0.57 when assessing the LA label design can be noted compared to 0.32 when assessing the HCD label design.

**Table 5:** HEART calculations of AHEP—label design assessed as the EPC 32

Generic task type = G Nominal human error probability = 0.002			
EPC	HEART affect multiplier	The assessed proportion of impact	Assessed EPC effect
2. A shortage of time available for error detection & correction	11	0.9	((11–1) × 0.9) + 1 = 10
17. Little or no independent checking or testing of output	3	0.86	((3–1) v 0.86) + 1 = 2.7
39. Distraction/Task Interruption	4	0.9	((4–1) v 0.9) + 1 = 3.7
32. Inconsistency of meaning of displays and procedures	3	0.91 (LA Label) 0.3 (HCD Label)	((3–1) × 0.91) + 1 = 2.8 ((3–1) × 0.3) + 1 = 1.6
Total assessed EPC effect	LA label	= (10 × 2.7 × 3.7 × 2.8) = 283.8	
	HCD label	= (10 × 2.7 × 3.7 × 1.6) = 161.0	
**Assessed human error probability**	**LA label = (0.002)** × **283.8 = 0.57** **HCD label = (0.002)** × **161.0 = 0.32**		

The relative percentage contribution to unreliability (RPCU) for each EPC was calculated (i.e. their gross effect on the human error probability). Results using EPC 6 and EPC 32 for label design assessment are presented in Tables 6 and 7, respectively. Table 6 shows a percentage of contribution of 29% when the LA label design was assessed, which was reduced to 13% for the assessment of the HCD label. In turn, Table 7 shows a reduction from 15 to 9% in the percentage of contribution to the HEP when assessing the HCD label.

**Table 6:** RPCU for each EPC—label design assessed as the EPC 6

EPC	Contribution to unreliability	
	LA label	HCD label
2. A shortage of time available for error detection & correction	43%	53%
17. Little or no independent checking or testing of output	12%	14%
39. Distraction/Task Interruption	16%	20%
6. A mismatch between an operator's model of the world and that of a designer	29%	13%

**Table 7:** RPCU for each EPC—label design assessed as the EPC 32

EPC	Contribution to unreliability	
	LA label	HCD label
2. A shortage of time available for error detection & correction	52%	55%
17. Little or no independent checking or testing of output	14%	15%
39. Distraction/Task Interruption	19%	21%
32. Inconsistency of meaning of displays and procedures	15%	9%

## Discussion

This study has applied the HEART prospective risk analysis method to an intravenous medication administration to estimate the influence of label design. Using the HEART method has revealed the task complexity in drug administration. Similar to previous studies using HEART in healthcare [39, 40], most of the time and attention of participants were invested in identifying the generic task and relevant EPCs involved in the simulated scenario.

The generic task G was selected by the participants in this study to describe the administration of intravenous medication in the scenario studied. However, some participants raised concerns that the G-task did not entirely fit the task under study. A possible explanation might be that the group of nurses who volunteered for the study had a heightened awareness of medication safety issues. In line with this finding, Chadwick and Fallon [40] reported that task G was chosen in a previous study where a task of recording abnormal blood results was analysed. However, participants in their study also considered that the task description did not fully match the task under analysis.

Regarding the most significant EPCs influencing human reliability in the analysed scenario, participants raised concerns regarding ‘A shortage of time available for error detection & correction’ (EPC 2). This EPC has a significant relative percentage contribution to unreliability (RPCU) ranging from 43 to 55%, according to the combination shown in Tables 6 and 7. This finding is consistent with that of Chadwick and Fallon [40], who reported the selection of this EPC in their study with a percentage contribution to unreliability result of 49%. Furthermore, it is crucial to consider that the risk of IV medication is more significant than administering medication by other routes. Because it is complicated to remove many medicines administered intravenously from the body, so few options are responding to help the patient, which may result in a fatal outcome for a newborn. The remedial measures from the HEART method suggest the need to give staff sufficient time to make critical decisions to avoid mistakes [35].

The ‘Little or no independent checking or testing of output’ (EPC 17) had a significant relative percentage contribution to unreliability ranging from 12 to 15%, according to the combination shown in Tables 6 and 7. Similar results were identified by Chadwick and Fallon [40], who reported a 14% in this value in a task recording abnormal blood test results. The HEART remedial measures emphasise the paramount independent checking of work when high reliability is needed [35]. Although double-checking is part of the required procedures of medication use, participants raised concerns that due to workload, only sometimes it is possible to perform. These concerns align with Kuitunen et al. [4], who suggest that failure in double-checking procedures is one of the leading systemic causes of medication errors.

‘Distraction and task interruption’ (EPC 39) were one of the biggest concerns of the nurses participating in this study. Given the strength of its multiplier (4) and the assessed proportion of the impact, this EPC has a significant RPCU ranging from 16 to 21%, which is lower than EPC 2 (shortage of time) but higher than the EPC 17 (lack of independent checking). No previous research in healthcare has identified the EPC 39, which can be explained by the fact that this EPC is a new one recently incorporated into HEART [46]. However, Chadwick and Fallon [40] describe how, in their study, interruptions (e.g. dealing with queries from doctors and patients, answering telephone calls) modify the task pacing of nurses (EPC 36). Moreover, interruptions are widely reported as a cause of medication administration errors [3, 50, 51].

In the study, the nurses perceived to be the most appropriate EPCs for evaluating label design were EPC 6 (a mismatch between an operator’s model of the world and that of a designer) and EPC 32 (inconsistency of meaning of displays and procedures). In a previous study using HEART in healthcare, the EPC 6 was related to poor system/human interface [39]. It could be assumed that LA labels, lack of contrast in the ampules, unclear labelling, and poorly designed packaging are examples of a mismatch between nurses’ and designers’ mental models. As mentioned before, pharmaceutical companies frequently do not consider a design with safety as a priority [14] because they assume practitioners are responsible for avoiding medication errors [15].

Our findings provide evidence to support the positive impact of the HCD labels on improving human reliability for medication safety. On the one hand, regarding a mismatch between nurses’ and designers’ mental models (EPC 6), results suggest a reduction of up to 60% according to the nurses’ assessment of the impact of label design on error probability (average assessed proportion of the impact). The assessed human error probability (AHEP) was reduced by approximately 50% (Table 4) when the HCD label was compared to the LA label. Another important finding was that the RPCU decreased by 44% from 29 (LA label) to 13 (HCD label) when label design was assessed (Table 6).

On the other hand, the positive impact of the HCD label on reducing human error may be observed because of inconsistency in the meaning of displays (EPC 32). In this case, results also suggest a reduction of up to 60% according to the nurses’ assessment of the impact of label design on error probability. The AHEP was reduced by approximately 56% (Table 5) when the HCD label was compared to the LA label. Again the RPCU decreased by 40% from 15 (LA label) to 9 (HCD label) when label design was assessed (Table 7). Consistently, the experienced nurses pointed out the risk in a high-stress situation, with limited time and resources to respond to the patient’s needs. These conditions might explain why there was a better score for safety medication using the HCD label.

This study has estimated the potential impact of label design in an IV medication administration process. However, confusion with look-alike medications has been pointed out as a systemic cause of errors not only in the medication administration tasks but rather in prescribing, dispensing, and storage tasks [4]. Therefore, the benefits of HCD labelling could also be assumed to reduce prescribing, dispensing, and storage errors. Furthermore, an HCD label reduces reading time and difficulty level [53], so it might help alleviate other systemic causes of medication errors, such as failure in double-checking procedures, work overload, and shortage of time. This example highlights the relevance of using HFE principles in designing medication labels, as suggested in previous research [18, 27, 53] and by international organisations [12, 16, 17]. Future research can utilise the findings of this study to estimate the impact of label design in other healthcare settings and processes of the medication system.

In addition, by applying the HEART method and going through the generic tasks and EPCs, the expert nurses could identify various factors and conditions influencing safety when administering medicine. The HEART method allowed quantification of the probability of error with these factors. However, it is important not to assess risks in isolation. We continue to set standards for healthcare based on what we imagine is correct, but we need to spend time understanding how the work is performed.

This study suffers from some limitations. First, our assessment was done in a simulated scenario and cannot predict the incidence of medication errors in the real world. However, our results are consistent with those observed in earlier studies. For instance, Endestad et al. [52] noted a reduction of two-thirds in errors when a redesigned package was used compared with original generic medications. Gupta et al. [53] found that redesigning the contrasting background of ampoules significantly reduced the reading errors of medications among resident physicians in a hospital. Similarly, in a controlled simulation study, Estock et al. [18] reported a significant reduction in incorrect medication selection from 63% using LA labels to 40% when using redesigned labels.

Another limitation was that the convenience sample included only a reduced number of nurses, which may induce a specific range of ideas that we cannot generalise to other contexts. Conversely, a strength of this study was that the group discussions and assessment included nurses with significant first-hand ‘sharp end’ experience in the clinical task under analysis. As mentioned above, applying the HEART method has been criticised as being too highly dependent on a single expert evaluator [40, 47]. Future work could involve more participants and a more comprehensive range of stakeholders (e.g. pharmacists).

## Conclusion

To the best of our knowledge, the current study is the first proactive analysis of human reliability analysis to estimate the influence of label design in the drug administration process using HEART. The technique used a small sample of experienced nurses to obtain first-hand insights into analysing this task. The results of this study provide evidence to support the positive impact of the HCD labels on improving human reliability for medication safety. The assessed human error probability was reduced by up to 56%, and the percentage of contribution to the unreliability of label design was consistently reduced by at least 40% in all the scenarios when the HCD label was evaluated. It must be noted that a shortage of time available for error detection and correction, no independent checking of outputs, and distractions were identified as factors that might increase human error probability (HEP).

This combination of findings supports the premise that the human-centred design of medication labels might improve medication safety, people’s well-being, and system performance. Future studies should examine the potential benefits of HCD labels in natural settings and include a more comprehensive range of stakeholders. Additionally, the complexity of factors contributing to administration errors suggests that a deeper understanding of systemic causes of medication errors is needed to improve patient safety significantly.

## Appendix

### HEART generic categories [48, 49]

**Table:**

Generic task	Proposed nominal human unreliability (5–95th percentile bounds)
(A) Totally unfamiliar, performed at speed with no real idea of likely consequences	041 (0.19–085)
(B) Shift or restore system to a new or original state on a single attempt without supervision or procedures	0.17 (0.02–1.0^a^)
(C) Complex task requiring high level of comprehension & skill	0.17 (0.05–0.6)
(D) Fairly simple task performed rapidly or given scant attention	0.06 (0.02–0.19)
(E) Routine, highly practised, rapid task involving relatively low level of skill	0.02 (0.005–0.09)
(F) Restore or shift a system to original or new state following procedures, with some checking	0.001 (0.00002–0.04)
(G) Completely familiar, well-designed, highly practised, routine task occurring several times per hour, performed to highest possible standards by highly motivated, highly trained & experienced person, totally aware of implications of failure, with time to correct potential error, but without the benefit of significant job aids	0.002 (0.0002–0.01)
(H) Respond correctly to system command even when there is an augmented or automated supervisory system providing accurate interpretation of system stage	0.00004 (0.000006–0.00009)

### HEART error-producing conditions [46, 49]

**Table:**

Error-producing conditions	Maximum predicted amount by which error probability changes from best to worst-case conditions
1. Unfamiliarity with a situation which is potentially important but which only occurs infrequently or which is novel	17
2. A shortage of time for error detection and correction	11
3. A low signal–noise ratio (when really bad)	10
4. A means of suppressing or over-riding information or features which is too easily accessible	9
5. No means of conveying spatial and functional information to operators in a form which they can readily assimilate – also known as Spatial and Functional incompatibility	8
6. A mismatch between an operator’s model of the world and that of a designer – also known as a Model Mismatch	8
7. No obvious means of reversing an unintended action – also known as Irreversibility	8
8. A channel capacity overload, particularly one caused by simultaneous presentation of non-redundant information – also known as channel overload	6
9. A need to unlearn a technique and apply one which requires an opposing philosophy – also known as technique unlearning	6
10. The need to transfer specific knowledge from task to task without loss- also known as knowledge transfer	5.5
11. Ambiguity in the required performance standards	5
12. A mismatch between perceived and real risk	4
13. Poor, ambiguous or ill-matched system feedback	4
14. No clear, direct, and timely confirmation of an intended action from the portion of the system over which control is to be exerted	3
15. Operator inexperience (e.g., a newly-qualified tradesman but not an “expert”)	3
16. An impoverished quality of information conveyed by procedures and person/person interaction	3
17. Little or no independent checking or testing of output	3
18. A conflict between immediate and long-term objectives	2.5
19. No diversity of information input for veracity checks	2.5
20. A mismatch between the educational achievement level of an individual and the requirements of the task	2
21. An incentive to use other, more dangerous procedures	2
22. Little opportunity to exercise mind and body outside the immediate confines of a job	1.8
23. Unreliable instrumentation (enough that it is noticed)	1.6
24. A need for absolute judgements that are beyond the capabilities or experience of an operator	1.6
25. Unclear allocation of function and responsibility	1.6
26. No obvious way to keep track of progress during an activity	1.4
27. A danger that finite physical abilities will be exceeded	1.4
28. Little or no intrinsic meaning in task	1.4
29. High level of emotional stress	2
30. Evidence of Ill-health among operatives, especially fever	1.2
31. Low workforce morale	1.2
32. Inconsistency of meaning of displays and procedures	3
33. A poor or hostile environment (below 75% of health or life-threatening severity	2
34. Prolonged inactivity or repetitious cycling of low mental workload tasks (× 1.1 for first half-hour, × 1.05 for each hour thereafter)	1.1
35. Disruption of normal work-sleep cycles—1.2 per 24 h of sleep lost	1.2
36. Task pacing caused by the intervention of others	1.06
37. Additional team members over and above those necessary to perform the task normally and satisfactorily—1.2 per additional person	1.2
38. Age of personnel performing recall, recognition, and detection tasks—1.16 for every ten years for ages 25 to 85 years (Mentally competent)	1.16
39. Distraction/Task Interruption	4
40. Time-of-Day- 2.4 from diurnal high arousal to diurnal/low arousal	2.4

## Declarations

### Confidentiality and information custody

The researchers guarantee information confidentiality and custody by not using it differently as an academic one.
